# Unraveling exosome-mediated cancer therapy resistance: pathways and therapeutic challenges

**DOI:** 10.1186/s43046-025-00289-9

**Published:** 2025-05-01

**Authors:** Sandip Sonwane, Umesh Telrandhe, Nikhita Chambhare, Sunita Vaidya

**Affiliations:** Datta Meghe College of Pharmacy, DMIHER (DU), Sawangi, Wardha, Wardha, India

**Keywords:** Exosomes, Cancer therapy resistance, Tumor microenvironment, Strategies, Challenges

## Abstract

Extracellular vesicles (EVs) have emerged as key cell-to-cell communication mediators and play significant roles in both physiological and pathological processes. In EVs, exosomes represent a distinct subpopulation of EVs that have been found to be involved in cancer initiation and therapeutic resistance. Exosomes transfer a diverse spectrum of molecular cargos that have significant effects on the tumor microenvironment (TME), thereby enabling cancer initiation, metastasis, and therapeutic resistance. Exosomes have recently been of interest in cancer therapy due to their role as important mediators of treatment resistance. The exosomal molecular content—proteins, miRNAs, and lncRNAs—allows exosomes to perform functions including drug efflux and detoxification, cell death pathway modulation, induction of epithelial-to-mesenchymal transition (EMT), and suppression of the immune system. In addition to facilitating immune and stromal cell interactions, exosomes cause extracellular matrix remodeling and induce tumor heterogeneity, making it more difficult to respond to therapy. This review covers intricate roles of exosomes in cancer therapy resistance with regard to their biogenesis, molecular content, and functional impact in the TME. Along with this, we also discuss new therapeutic strategies to overcome exosome-mediated resistance including utilizing exosome inhibitors, designed exosome therapy, and combination with conventional therapies. While exosomes hold promise in prediction and diagnosis through their biomarker function, their heterogeneous origins and cryptic functions make it difficult to target interventions. This review emphasizes that research on exosome-mediated pathways is urgently required to develop new therapeutic strategies that can improve cancer treatment outcomes.

## Introduction

Exosomes are small, membrane-bound EVs that have recently been recognized as key regulators of intercellular communication [1]. Nanosized vesicles, with diameters commonly in the range of 30–150 nm [2], are secreted by virtually all cell types into various biological fluids, including blood, urine, saliva, and even breast milk [3]. Exosomes originate from the inward budding of membranes within multivesicular bodies and are released into the extracellular space through the fusion of multivesicular bodies (MVBs) with the plasma membrane [4]. Although exosomes have long been considered cellular waste, interest in the important biological role they play in facilitating critical processes is increasing. They mediate the intercellular transfer of proteins, lipids, and nucleic acids, including DNA, RNA, and microRNAs, and thus participate in intercellular communication [5]. Exosomal molecular cargos contribute to cancer development through modulation of the tumor microenvironment, induction of cell division, promotion of drug resistance, and assistance with metastasis. The miRNAs, proteins, and lncRNAs present in exosomes can suppress immune response, enhance angiogenesis, and induce EMT [6]. These functions are thought to have a strong impact on physiological and pathological conditions, such as immune responses, inflammatory pathways, and tumor development. Until recently, many studies have focused on the function and mechanisms of exosomes, as their diverse roles in various biological contexts, some of which have been linked specifically to cancer [7]. Exosomes are also considered important components in the biology of cancer and act as paracrine mediators between the tumor and stroma. These exosomes are exploited by cancer cells to favor the growth, invasion, and metastatic capability of tumor cells [8]. Through these vesicles, the oncogenic factors of tumor cells are transferred to the surrounding tumor microenvironment and contribute to the metastasis of cancer [9]. These effects are mediated through their ability to change the behavioral properties of both near and distant cells. The contents of exosomes generally reflect the host cells from which they are derived, with their cargo typically bearing various classes of molecules, including proteins, RNA, and lipids, which have functional roles in altering cellular behaviors [10]. For example, exosomes secreted by tumor cells carry oncogenic proteins and RNA that can functionally reprogram recipient cells toward promoting tumor development. In addition, exosomes can contribute to angiogenesis, through which tumors receive the nutritive and oxygen needs that are necessary for tumor growth. They can also suppress immune responses, enabling tumors to evade detection and destruction by the body’s immune system [11].

Among the various key roles that exosomes play in cancer biology, their participation in the metastatic process plays a leading role [12]. Metastasis is a multistep process by which cancer cells from a primary tumor site migrate through the body to reside in distant organs and form secondary tumors [13]. In this process, exosomes may facilitate the metastatic niche by preparing a good environment in distant tissues for arriving cancer cells. This is accomplished by the transfer of prometastatic signals through exosomes to distant cells, which may lead to changes in the extracellular matrix, increased vascular permeability, and the survival and colonization of tumor cells [14]. Exosomes promote the invasion of tumor cells via cleavage of the extracellular matrix, which is regulated by matrix metalloproteinases [15].

In addition to promoting metastasis, exosomes have also been implicated in drug resistance, which is one of the major challenges of cancer therapy. Resistance to therapy may occur through a variety of mechanisms, including genetic mutation, epigenetic modification, and altered conditions of the tumor microenvironment [16]. Exosomes have been demonstrated to be involved in both intrinsic and acquired resistance by transferring drug resistance-related molecules, including drug efflux pumps, antiapoptotic proteins, and microRNAs, from resistant cells to sensitive cells [17]. This may protect cancer cells from the effects of cytotoxic chemotherapy, thus leading to treatment failure and disease recurrence. For example, exosomes derived from drug-resistant tumor cells have been shown to carry multidrug resistance proteins, including P-glycoprotein, which can be transferred to drug-sensitive cells, rendering them resistant to chemotherapy [18]. Exosomes may also alter the tumor microenvironment in ways that favor resistance, such as by inducing the expression of survival pathways or by repressing immune responses that would otherwise target and destroy cancer cells [19].

Given the critical role of exosomes in cancer progression, metastasis, and resistance to therapy, their investigation has become a prerequisite for any new therapeutic approach. Among these, one of the most promising areas involves the use of exosomes as diagnostic and prognostic biomarkers. Because exosomes are released into body fluids and carry molecular signatures from their parent cells, they represent a minimally invasive means of monitoring disease [20]. For instance, exosomal RNA and proteins can be analyzed for the presence of specific mutations or alterations associated with cancer for early diagnosis, tracking disease progression, or predicting response to treatment [21]. Other approaches may involve the use of exosomes as therapeutic targets [22].

Cancer therapy resistance is of utmost importance in research on cancer treatment. Resistance to therapy is one of the main causes of the poor results observed in cancer treatment. Chemotherapy and radiotherapy, as well as some forms of targeted therapies, have their own way of failing when cancers develop resistance [23]. Often, cancers that present an initial good response to therapies over time usually progress to the development of resistance before relapsing, resulting in highly poor outcomes [24]. Understanding the mechanisms of resistance to therapy is important for overcoming this challenge and further improving the efficacy of treatments against cancer. Exosomes are increasingly recognized for their role as key mediators of resistance to therapies, not only by transferring resistance factors but also by reprogramming the tumor microenvironment and immune response [25]. For example, exosomes may induce an immunosuppressive environment that promotes the immune escape of cancer cells and further contributes to resistance [26]. Research on exosomes and their functions in relation to therapy resistance aims to identify new targets for treatment and develop methods for overcoming resistance. Another approach may be the combination of standard therapeutic procedures with those affecting either the production or functions of exosomes to hinder the spread of resistance factors [27]. Other methods may involve the use of exosomes as drug carriers, taking advantage of their natural ability to shuttle between cells. Because they can be loaded with a variety of materials, ranging from chemotherapeutic agents to other therapeutic molecules, exosomes might be bioengineered to target selected cancer cells and thereby overcome resistance and decrease side effects [28].

## Biogenesis and function of exosomes

### Formation of exosomes

**Fig. 1:**
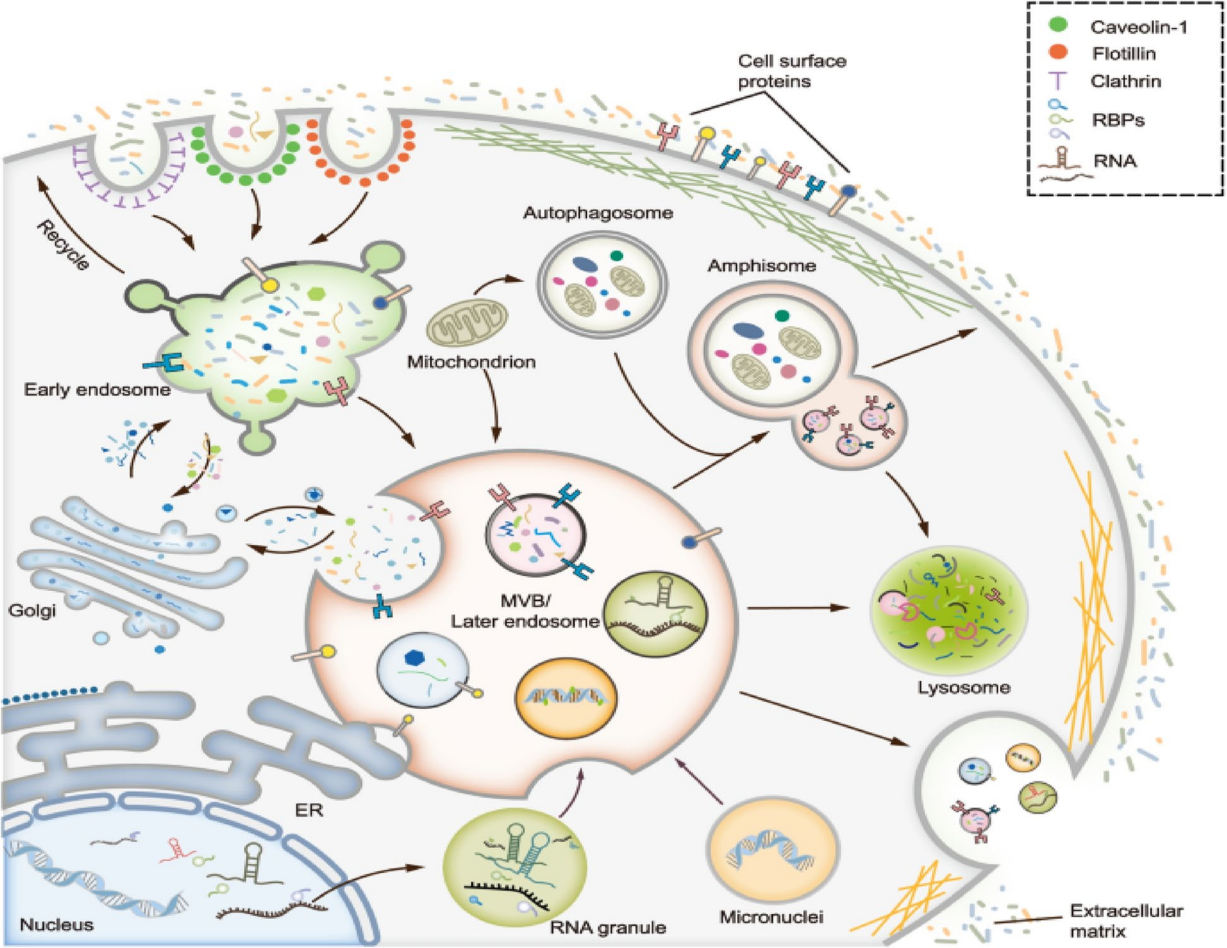
The process of exosome biogenesis. Exosomes were originally derived from endocytosis and mostly interact with numerous other organelles, facilitating cargo sorting, including proteins, RNAs, DNAs, and lipids. Eventually, mature MVBs can fuse with either lysosomes for degradation or the plasma membrane to release intraluminal vesicles as exosomes. Additionally, MVBs may form fusions with the autophagosome to form an amphisome; MVBs can further be degraded or secrete an exosome [31]

Exosome formation is a series of highly coordinated events within the endosomal network, representing a highly specialized system implicated in sorting, trafficking, and turnover of cellular entities [29]. Biogenesis of the exosome starts with budding toward the inside of the plasma membrane, after which early endosomes are formed. Early endosomes mature into late endosomes, also referred to as multivesicular bodies or MVBs, which are characterized by the accumulation of intraluminal vesicles within their lumen. These intraluminal vesicles represent the precursor structures of exosomes [30]. The summary of biogenesis of exosome has been provided in Fig. 1.

#### Initiation: formation of early endosomes

The process of exosome biogenesis starts at the plasma membrane, where select molecules are internalized by endocytosis into the cell through clathrin-coated vesicles or other modes of endocytosis [32]. The vesicles that form in the plasma membrane fuse with early endosomes, which act as sorting compartments for the internalized material. Within these sorting compartments, proteins, lipids, and other molecules are selectively sorted for degradation, recycling, or incorporation into ILVs [33].

#### Maturation of endosomes

These structures undergo successive maturation steps, changing them from early endosomes into late endosomes, also termed multivesicular bodies or MVBs [34]. One of the dramatic morphological changes in these steps is the inward budding of the endosomal membrane, leading to the creation of intraluminal vesicles or ILVs. Specific proteins, lipids, and RNAs destined for exosomal secretion are selectively internalized into such ILVs [35].

Sorting cargo into ILVs is directed by two main pathways: the ESCRT-dependent pathway and the ESCRT-independent pathway. The ESCRT-dependent pathway relies on the sequential action of protein complexes, including ESCRT- 0, ESCRT-I, ESCRT-II, and ESCRT-III, for the capture and budding vesicle formation of ILVs. Recognition of specific cargo in this complex occurs via ubiquitinated cargo protein molecules through the ESCRT- 0 complex, whereas ESCRT-I and ESCRT-II serve to further drive bending and membrane invagination via force induction. ESCRT-III, in conjunction with accessory proteins, promotes vesicle scission to release ILVs into the lumen of the MVBs [36].

In contrast, the ESCRT-independent pathway is dependent on the use of lipid rafts, tetraspanins, or ceramide-enriched microdomains to drive ILV formation. Among these, the bioactive lipid ceramide is particularly important for inducing the membrane curvature that drives ILV budding [37].

### Fusion of MVBs with the plasma membrane

Once MVBs mature and are loaded with ILVs, they are capable of fusing with lysosomes for degradation of their contents or with the plasma membrane, releasing ILVs in the form of exosomes. The latter process relies on the action of several proteins, including Rab GTPases such as Rab27a and Rab27b, SNARE proteins, and other accessory molecules. Once MVBs merge with the plasma membrane, ILVs are released as exosomes, which can be internalized by surrounding cells or travel through bodily fluids to interact with distant tissues [38–40].

#### Exosome secretion

Exosome secretion is a well-regulated process that is dependent on both intracellular and extracellular signals [41]. The rate of exosome secretion in cells depends on physiological conditions, environmental stress, or pathological stimuli, guaranteeing the delivery of their molecular cargo to the target cells. Some regulatory factors of exosome secretion include intracellular Ca2 +, hypoxia, and oxidative stress, and the associated signaling pathways include the MAPK/ERK and PI3 K/AKT pathways [42].

##### Calcium-dependent secretion

Intracellular calcium is a key regulator of exosome secretion. Increased intracellular calcium facilitates the fusion of MVBs with the plasma membrane and, thus, the release of exosomes. This calcium-dependent exosome secretion has often been observed in neurons and other excitable cells, where changes in intracellular calcium are a common feature [43, 44].

##### Hypoxia- and stress-induced secretion

Exosomes have also been shown to increase their production and release due to hypoxia or stressful oxidative conditions. Hypoxic conditions in cancer cells increase exosome secretion, which might be related to tumor invasion and metastasis. Stress-induced extracellular vesicle release may even further contribute to tissue repair mechanisms and cellular adaptation by shuttling stress response proteins and RNAs between these cells [45, 46].

#### Molecular cargo of exosomes

**Fig. 2:**
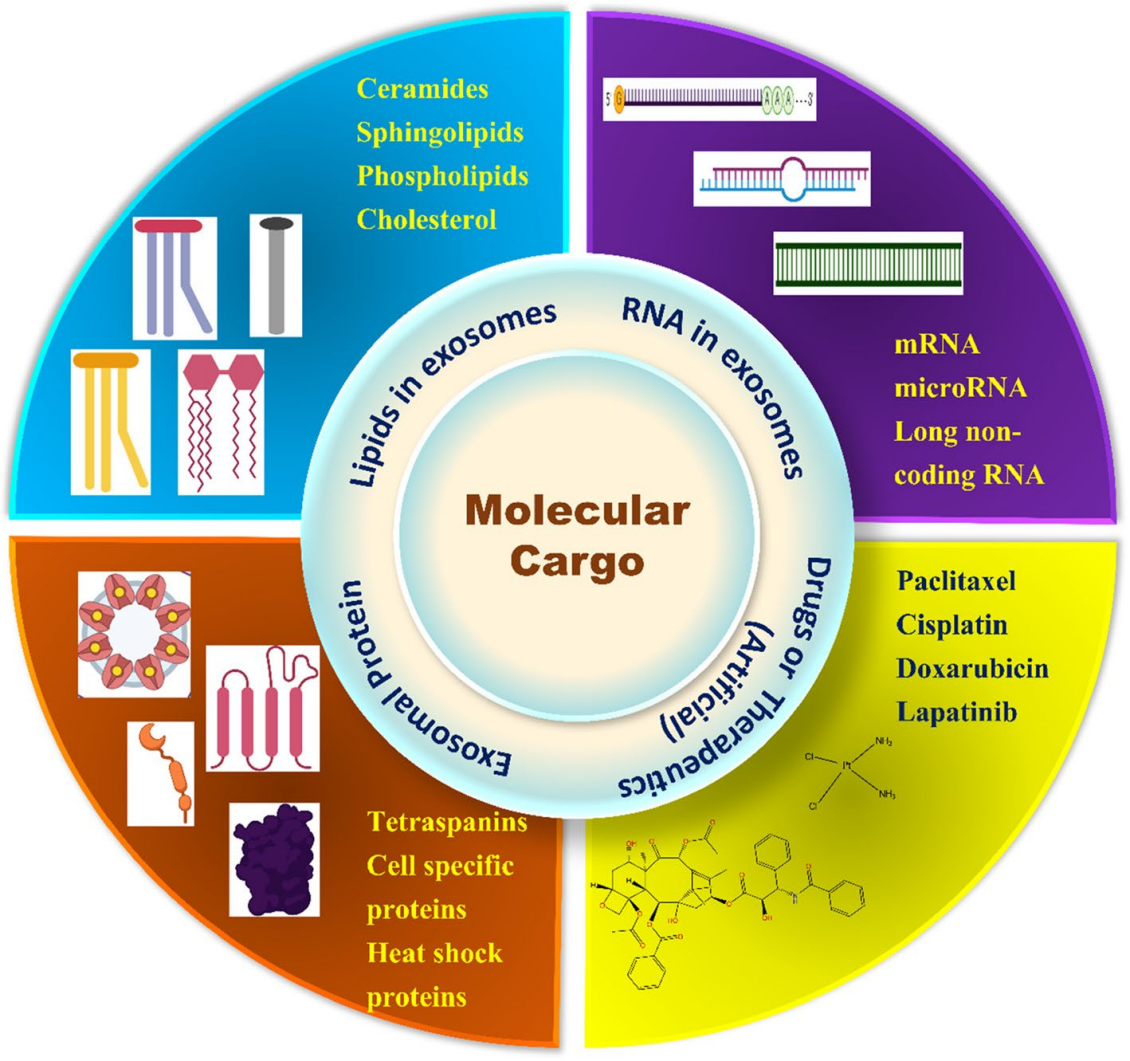
Overview of the molecular cargo in exosomes

Exosomes are enriched in a wide array of molecular cargo, including proteins, lipids, and nucleic acids such as RNA. This cargo is selectively packaged during exosome biogenesis and reflects the physiological state of the parent cell. The molecular content of exosomes can influence the behavior of recipient cells, making exosomes powerful mediators of intercellular communication. The subsequent sections describe the key components of exosomal cargo [47–49]. The overview on molecular cargo of exosomes has been provided in Fig. 2.

##### Exosomal proteins

Exosomes carry various proteins that reflect both the common molecular machinery involved in the biogenesis of exosomes and cell-type-specific proteins. Some proteins are universal among all exosomes, whereas others are specific either to the cell of origin or to the physiological conditions under which the exosomes are formed.

##### Tetraspanins

The most abundant protein families within exosomes include tetraspanins such as CD9, CD63, CD81, and CD82. These membrane-spanning proteins are implicated in the formation of exosomes, cargo sorting, and cellular targeting. Tetraspanins also serve as biomarkers for exosome isolation and characterization [50].

##### ESCRT-related proteins

Owing to the role of the ESCRT machinery in exosome biogenesis, many ESCRT components are common in exosomes. This includes proteins such as Alix, TSG101, and VPS4, which have implications for the budding and release of ILVs. ESCRT-related proteins often serve as markers of exosomal purity in experimental procedures [51, 52].

##### Heat shock proteins

A general hallmark of exosomes consists of the heat shock proteins HSP70 and HSP90. Heat shock proteins are the most active proteins that perform their functions during responses to cellular stress; the participation of heat shock proteins has been shown to involve several processes related to proteins, such as folding, stabilization, and trafficking. Thus, another major potential function of heat shock proteins in exosome transport is protection during exosomal transport via the extracellular space [53].

##### Cell-specific proteins

In addition to common exosomal proteins, exosomes also carry cell-specific proteins that reflect the physiological or pathological state of the parent cell. For example, exosomes derived from tumor cells often contain oncogenic proteins, including epidermal growth factor receptor (EGFR), which may influence cancer progression. Similarly, exosomes secreted by immune cells may carry immune-regulatory proteins such as MHC class I and II molecules, cytokines, and costimulatory proteins that influence immune responses [54].

##### RNA in exosomes

Exosomes are enriched in various species of RNA, including mRNAs, microRNAs, and other noncoding RNAs. These species of RNA are selectively loaded into exosomes during biogenesis and can be transferred to recipient cells, where they influence gene expression and cellular function [55, 56].

##### Messenger RNA

This means that the entry of exosomal mRNAs into recipient cells can be translated into proteins, thereby changing the profile of proteins in recipient cells. A few reports have indicated that exosomes can deliver full-length mRNA sequences that are functionally expressed in recipient cells, indicating that one mechanism for modulating activity across cells between tissues involves the transfer of mRNAs through exosomes [57].

##### microRNA

One of the best-studied RNA classes in exosomes is represented by microRNAs, small noncoding RNAs that regulate gene expression through targeting for degradation or translational repression of certain mRNA transcripts. miRNAs contained in exosomes can modulate the activity of critical pathways and change recipient cell behavior. For instance, exosomes secreted from tumors express oncogenic miRNAs that enhance tumor growth and metastasis, whereas immune cells may carry exosomal miRNAs related to immune regulation [58].

##### Long noncoding RNAs

Long noncoding RNAs constitute another class of RNA molecules present in exosomes. Compared with that of miRNAs, much less is known about the functional aspects of lncRNAs. However, it is surmised that they participate in chromatin remodeling at the level of the regulation of transcription and posttranscriptional modifications. lncRNAs have the potential to modulate the expression of genes and impose epigenetic changes upon recipient cells [59].

##### Lipids in exosomes

Exosomes are enriched in certain lipid species that provide structural integrity, cargo sorting, and biological activity. The composition of lipids in exosomes differs from that in the plasma membrane of the parent cell and is an important player in both exosome formation and functionality [60].

##### Sphingolipids and ceramides

Sphingolipids, to which ceramide belongs, are critical factors in the budding of ILVs during the biogenesis of exosomes. Indeed, ceramide promotes membrane curvature and vesicle budding and is thus absolutely necessary for the production of exosomes. Exosomes contain bioactive sphingolipids that may contribute to cell signaling and even apoptosis in recipient cells [61].

##### Cholesterol

Another essential lipid of exosomes is cholesterol. Cholesterol is stable in the membrane of exosomes and confers rigidity to the structural integrity of the vesicle. Additionally, cholesterol-rich microdomains may play a role in the selective sorting of membrane proteins during biogenesis in exosomes [62].

##### Phospholipids

Among others, exosomes contain phospholipids such as phosphatidylserine, phosphatidylcholine, and phosphatidylethanolamine. Phosphatidylserine may be present on the outer leaflet of the exosomal membrane and usually acts as a recognition signal toward recipient cells. The difference in the type of lipids within the exosomes may determine the mode by which these vesicles are internalized and interact with the recipient cells [63].

## Mechanism of exosome-mediated communication

**Fig. 3:**
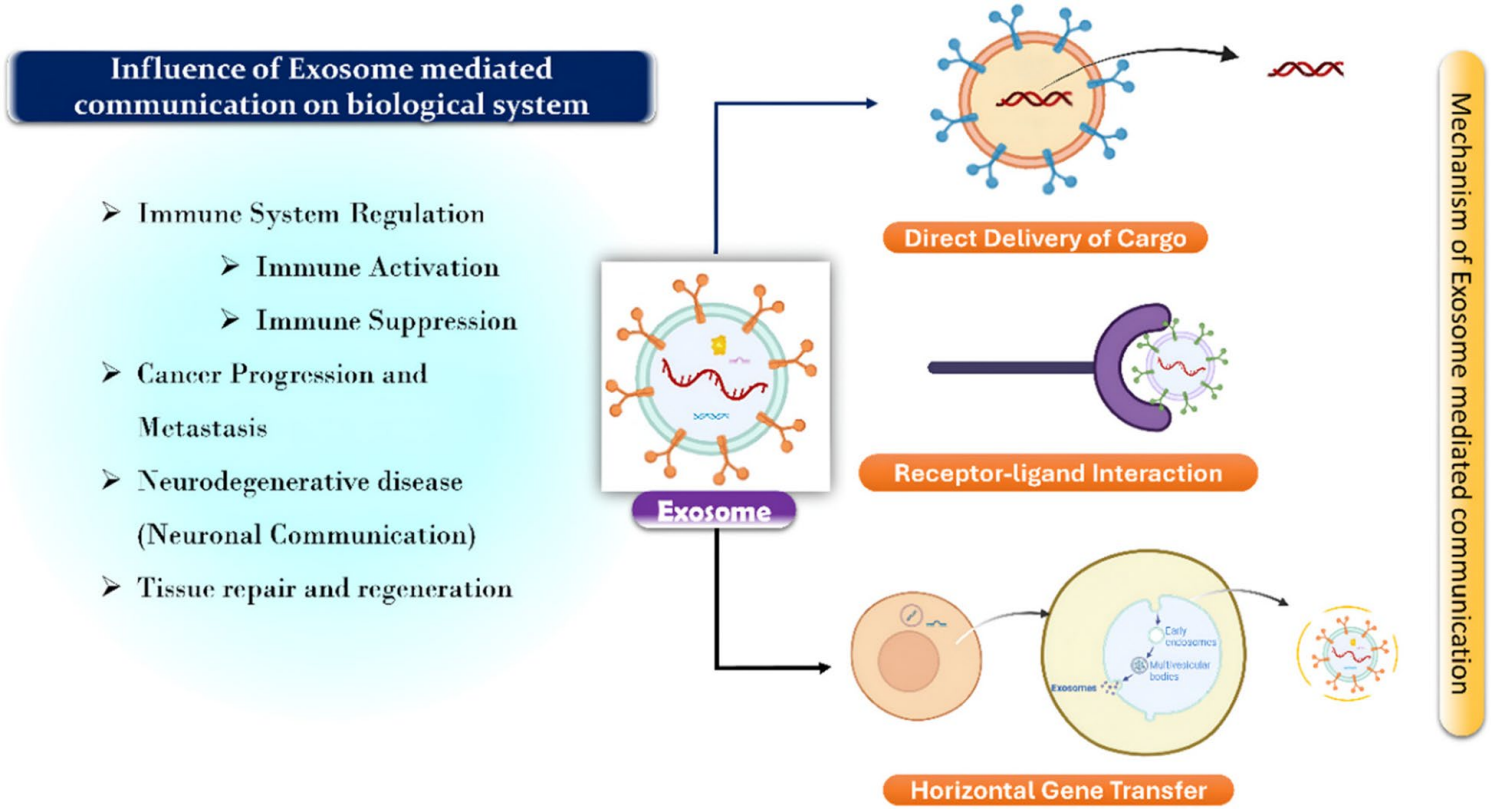
Schematic representation of the mechanism of exosome-mediated communication and its influence on biological systems

Exosome-mediated communication is a complex form of intercellular signaling that is at the heart of many physiological and pathological processes [64]. Nanosized extracellular vesicles (exosomes) act as transporters of various bioactive molecules, including proteins, RNA species such as mRNAs, miRNAs, long noncoding RNAs, lipids, and other molecules that are selectively packaged during biogenesis [65]. Their major mode of action includes the delivery of cargo into recipient cells, thereby modulating gene expression, signal transduction, and metabolic activity [66]. As illustrated in Fig. 3, exosome-mediated communication significantly impacts biological processes. Exosome cargo selection is a regulated process that is determined by cell type specificity, environmental conditions, and cell machinery. The key mechanisms involved include ESCRT-dependent and ESCRT-independent mechanisms. ESCRT-dependent mechanisms sort proteins tagged with ubiquitin [67], while ESCRT-independent mechanisms triggered by ceramides and lipid rafts sort proteins [68]. RNA-binding proteins like hnRNPA2B1 selectively load miRNAs to target their delivery [69]. RAB GTPases and cytoskeletal proteins control trafficking and release of cargoes and play significant roles in vesicle transportation [70]. The selection of cargoes is cell-type-specific; immune cells release immune-modulating factors [71], while cancer cells release oncogenic proteins and pro-metastatic RNAs [72]. Environmental conditions like hypoxia, genetic mutations, and pathological conditions also modulate exosome cargoes to enhance exosome complexity [73]. The specificity of exosomes makes them significant in biomarker discovery, targeted therapy, and understanding therapy resistance. In addition, exosomes can induce receptor‒ligand interactions on the surface of recipient cells, thus activating intracellular cascades without internalization [74]. For instance, tumor-derived exosomes employ surface integrins to manipulate adhesion and migration. Exosomes are also mediators of horizontal gene transfer, transporting genetic materials into recipient cells to alter their behavior. This is a well-recognized feature in the development of cancer, where exosomes transport oncogenes or drug resistance genes [75].

These nanostructures either act as activators or suppressors in immune regulation. Antigen-presenting cell-derived exosomes, which express MHC and costimulatory molecules, play an important role in the T-cell activation that characterizes adaptive immune responses [76]. In contrast, tumor-derived exosomes, which are rich in the immunosuppressive molecule PD-L1, suppress cytotoxic T cells to promote immune escape. Similarly, exosomes derived from regulatory T cells also carry immune suppressive cytokines, including TGF-β and IL- 10, which contribute to immune suppression in chronic inflammatory diseases and cancer [77].

In oncology, exosomes are critical for modulating the tumor microenvironment and promoting metastasis. Tumor cell-derived exosomes reprogram normal fibroblasts within the tumor stroma into malignant fibroblasts, so-called cancer-associated fibroblasts, augmenting tumor growth and supportive neoangiogenesis via cargo transfer of proangiogenic factors such as VEGF into endothelial cells [78].

In neurodegenerative diseases, exosomes spread the pathological proteins amyloid-β, tau, and α-synuclein, accelerating the progression of diseases such as Alzheimer’s disease and Parkinson’s disease. These exosomes spread misfolded proteins across neurons and amplify neuroinflammation, leading to further neuronal damage. Microglia-derived exosomes contribute to either neuroinflammation or neuroprotection, depending on their cargo, highlighting their dual role in neurodegenerative conditions [79–81].

Exosomes are involved in tissue repair via regeneration mechanisms. Importantly, MSC-derived membrane-derived exosomes effectively promote tissue repair in heart, kidney, and head injuries.

Growth factors, various cytokines, and critical miRNAs decrease with the inflammatory response while accelerating cell survival and angiogenesis signals. For example, mechanisms such as inhibiting cardiomyoblast apoptosis and promoting vascular regeneration are observed in cardiac injury-induced insults [82].

## Mechanisms of exosome-mediated resistance to therapy

### Drug efficacy

In most situations where chemoresistance arises, one of the mechanisms of resistance is exosome-mediated drug efflux; this process is most often involved in a number of multidrug-resistant cases. It typically contains exosomal packaging and the export of chemotherapeutic agents outside cancer cells. P-gp and other well-known ATP-binding cassette transporters associated with MDR can often be found on the membranes of exosomes [83]. These proteins are involved in the active efflux of drugs from the cytoplasm into the lumen of exosomes, which are then released into the extracellular space. Once released, these exosomes can be internalized by surrounding cells, thus propagating drug resistance within the tumor microenvironment [84].

For example, in the case of breast cancer, exosomes carry P-gp, which is responsible for the active efflux of chemotherapeutic agents such as doxorubicin, thus reducing intracellular drug accumulation. Some drug transporters associated with exosomal membranes effectively lower the intracellular concentration of chemotherapeutic agents, thus ensuring the survival and proliferation of cancer cells despite ongoing drug treatment [85, 86].

### Drug detoxification

In addition to drug efflux, other detoxification processes involve exosomes neutralizing the cytotoxic effects of chemotherapeutic agents through packaging and exporting detoxifying enzymes and proteins [87]. Glutathione S-transferases are detoxifying enzymes that neutralize ROS and other noxious metabolites generated by chemotherapy. The exosomes of resistant cancer cells are frequently loaded with GSTs, which neutralize the cytotoxic byproducts of chemotherapy, hence rendering the treatments less effective [88].

Other aspects of exosomal detoxification include the removal of damaged or oxidized proteins that are produced as a result of chemotherapy-induced oxidative stress. The export of such proteins within exosomes enables cancer cells to maintain their intracellular homeostasis, further enhancing their resistance to therapy [89].

### Altered apoptotic pathways

Programmed cell death, or apoptosis, is the major mechanism through which most anticancer therapies exert their effects. Indeed, chemotherapy, radiotherapy, and targeted therapies are known to induce apoptosis through the activation of proapoptotic signaling pathways or the inhibition of antiapoptotic pathways. However, exosomes may modulate apoptotic signaling as a means to promote therapeutic resistance by inhibiting apoptosis in cancer cells.

#### Anti-apoptotic proteins in exosomes

Drug-resistant cancer cells often secrete exosomes that carry antiapoptotic proteins, protecting them from chemotherapy-induced cell death. Survivin, Bcl- 2, and XIAP, for example, are common in exosomes derived from resistant tumor cells. These proteins inhibit important components of the apoptotic machinery involved in the execution phase of apoptosis, namely, caspases. By delivering these antiapoptotic factors to surrounding cells, exosomes are able to spread resistance to therapy within the tumor microenvironment and enable cancer cells to escape apoptosis [90].

In some tumor types, including leukemia and lymphoma, exosome-mediated transfer of antiapoptotic proteins has been shown to provide resistance against therapies directed against the intrinsic pathway of apoptosis. For example, exosomal Bcl- 2 is capable of suppressing the mitochondrial-mediated apoptosis pathway, which, very often, is induced by chemotherapeutic drugs [91].

### MicroRNAs and inhibition of apoptosis

Moreover, in addition to proteins, exosomes carry miRNAs that can modulate apoptotic signaling by either degrading or repressing specific mRNAs. Specific miRNAs, such as miR- 21 and miR- 155, which were previously reported to repress proapoptotic genes, are relatively enriched in exosomes derived from drug-resistant cancer cells. These miRNAs might repress the expression of proapoptotic factors such as PTEN (phosphatase and tensin homolog) and Fas ligand to prevent the activation of apoptosis, thus favoring survival upon chemotherapy treatment [92]. Exosomes are known to transfer antiapoptotic miRNAs, suggesting a role in resistance to several therapies that target pivotal regulators of cell survival/apoptosis, including both the PI3 K/AKT and MAPK/ERK signaling pathways. By modulating the expression of such crucial signaling molecules, exosomes are able to sustain tumor cell viability even under exposure to apoptotic stimuli [93].

### Epithelial–mesenchymal transition

The epithelial-to-mesenchymal transition can be explained as follows: a biological process including some epithelial cells with characteristic cell polarity and adhesion results in an enhanced mesenchymal role, enhanced cellular migration ability, invasion, and other cells. Owing to the ability of various neoplasias to promote EMT, therapies related to development resistance are typically available owing to the more aggressive malignant nature and/or stem characteristics provided by these cells for the adoption of such types of expression by cancerous cells of other phenotypes. Studies have elucidated the role and significant contribution of exosomes that promote the process of EMT development, resulting in anticancer treatments associated with drug resistance to therapeutic agents [94].

#### Exosomal transfer of EMT-inducing factors

Exosomes from EMT-expressing tumor cells are packaged with proteins, miRNAs, and other molecules that directly propagate the EMT phenotype to surrounding cells. For example, exosomes have often been reported to carry TGF-β, a cytokine that potently induces EMT. Indeed, the activation of transcription factors such as Snail, Slug, and Twist downstream of TGF-β signaling normally mediates E-cadherin repression via the expression of mesenchymal markers, including N-cadherin and vimentin. These exosomes may induce phenotypic transformation in surrounding cells by transferring these EMT-inducing factors, thus increasing resistance to chemotherapy and promoting metastasis [95, 96].

Exosomal miRNAs also induce EMT. Notably, the expression of miR- 200 family members, known suppressors of EMT, is often downregulated in exosomes derived from drug-resistant cancer cells, whereas the expression of miRNAs such as miR- 21 and miR- 27a, which promote EMT, is enriched. These factors could suppress genes maintaining the epithelial phenotype and hence further drive EMT to contribute to therapy resistance [97, 98].

#### EMT and cancer stem cells

EMT is closely associated with the features of CSCs, which are responsible for their resistance to chemotherapy, radiation, and targeted therapies. CSCs are capable of self-renewal and differentiation under unfavorable conditions that are imposed by anticancer therapies. Specifically, exosomes from EMT-driven cancer cells are capable of transferring promoting factors for CSCs, including Wnt proteins and Hedgehog signaling molecules, to recipient cells, resulting in an increase in the population of CSCs. This, in turn, enhances resistance to therapy by increasing the number of cells that can bypass the treatment and cause recurrence [99, 100].

### Immune modulation and evasion

The immune system is the most important barrier to tumor cells, but tumors often develop a way of escaping immune surveillance, thus allowing them to evade the destruction effect exerted by immune cells. Exosomes are considered key mediators of immune modulation and evasion within the tumor microenvironment, thus promoting the survival of cancer cells through immune dampening.

#### Suppression of immune cell activity

Tumor-derived exosomes often express immune suppressive molecules, impeding the function of immune effector cells such as cytotoxic T cells and NK cells. For instance, exosomes that bear PD-L1 are capable of interacting with the receptor PD- 1 on T cells to inhibit their activation and foster an immunosuppressive state. This is particularly important in the context of immune checkpoint blockade therapies, where tumors can secrete exosomes as a means of bypassing the action of immune checkpoint inhibitors, thereby creating a state of therapeutic resistance [101].

Exosomes transport immunosuppressive cytokines, including transforming growth factor-beta and interleukin- 10, which downregulate the activity of effector immune cells and promote the expansion of regulatory T cells. Regulatory T cells are involved in maintaining immune tolerance and preventing excessive immune responses; however, in the context of the tumor microenvironment, their activities may contribute to immune evasion by suppressing antitumor immune responses [102].

#### Immune tolerance induction

In addition to inducing immunosuppression, exosomes induce immune tolerance, which modulates the antigen-presenting function of immune cells such as dendritic cells and macrophages [103].

## Role of exosomal PD-L1 in immune checkpoint blockade therapy resistance

**Fig. 4:**
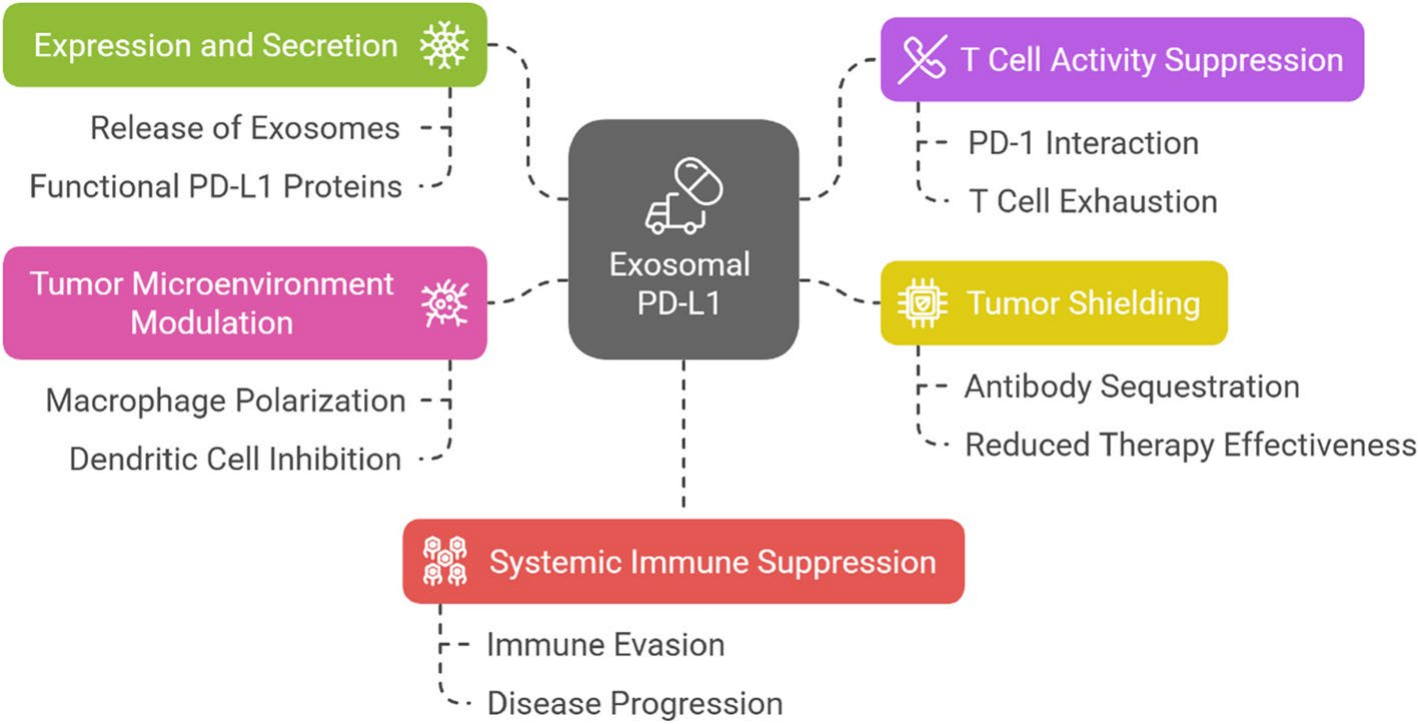
Schematic representation illustrating the role of exosomal PD-L1 in mediating resistance to immune checkpoint blockade therapy

Exosomal PD-L1 is a primary driver of immune checkpoint blockade (ICB) resistance through multiple mechanisms to dampen anti-tumor immunity. Tumor cells actively secrete exosomes that have loaded PD-L1 to replicate the immunosuppressive effect of membrane-bound PD-L1 and extend immune suppression to regions outside of the tumor microenvironment. In addition, exosomal PD-L1 acts as a decoy to bind to anti-PD- 1 antibodies and reduce their ability to neutralize tumor-bound PD-L1. Moreover, circulating exosomes that are positive for PD-L1 can cause systemic immune suppression to enhance metastatic cell survival in distant organs [104]. The exosomes dampen T cell receptor (TCR) signaling by interacting with PD- 1 receptors on T cells to cause T cell exhaustion and blunted immune response. Exosomal PD-L1 also modulates the tumor microenvironment by causing macrophage polarization to an immunosuppressive state and inhibiting dendritic cell maturation [105]. Collectively, these mechanisms play a critical role in ICB resistance and pose a key challenge to increasing therapeutic efficacy in cancer therapy. The role of exosomal PD-L1 in mediating resistance to immune checkpoint blockade therapy has been summarized in Fig. 4.

## Clinical relevance of exosome-mediated drug resistance

Exosome-mediated resistance in cancer is one of the greatest challenges in clinical oncology and makes treatment outcomes difficult, contributing to disease progression, recurrence, and metastasis. In cancer, exosomes mediate resistance to chemotherapy, radiotherapy, immunotherapy, and targeted therapy. This exosome-mediated resistance has profound clinical implications for cancer treatment strategies, diagnostic approaches, and therapeutic interventions.

### Impact on chemotherapy resistance

Chemotherapy is one of the cornerstones of cancer treatment, but the development of resistance often reduces its effectiveness [106]. Exosomes are known to play critical roles in this process by mediating the intercellular transfer of resistance-associated molecules. For instance, exosomes from drug-resistant cancer cells often carry ATP-binding cassette transporters, including P-glycoprotein, which pump chemotherapeutic drugs out of the cells, hence reducing drug accumulation and efficacy [107].

These exosomes may transfer ABC transporters to other drug-sensitive cancer cells in the neighborhood, thus conferring resistance across the TME. The transfer of microRNAs by exosomes is not beyond the cycle of processes leading to chemotherapy resistance. Specific microRNAs, including miR- 21 or miR- 155, which are common in exosomes derived from resistant cancer cells, downregulate the expression of several proapoptotic genes or upregulate the expression of these drug efflux transporters in the same recipient cellular elements, thus promoting their survival under chemotherapy [108]. Additionally, this may increase tumor heterogeneity, resulting in populations of resistant persistent cells even after treatments take place. Clinically, this leads to treatment failure, relapse, and the development of more aggressive tumors. For these reasons, managing cancers that are resistant to chemotherapy has become very difficult.

In a variety of tumor types, including breast cancer, ovarian cancer, and pancreatic cancer, exosomes are capable of mediating chemoresistance. As, in the case of breast cancer, exosomes bearing drug-resistant proteins such as HER2 and P-gp have been associated with resistance to conventionally used chemotherapeutic agents such as doxorubicin and paclitaxel. This exosomal transfer of resistance factors accounts for a poor prognosis and limits the efficacy of standard chemotherapy regimens [109].

### Impact of resistance to targeted therapy

Targeted therapies involving the inhibition of oncogenic signaling pathways have revolutionized cancer treatment. However, exosome-mediated resistance has emerged as a great barrier to their long-term success. Often, tumor cells secrete exosomes carrying components of targeted pathways to evade the effects of these therapies. E.g., in non-small cell lung cancer, exosomes expressing mutated forms of the epidermal growth factor receptor have been implicated in resistance to EGFR-targeted therapies, such as tyrosine kinase inhibitors [110]. In addition to harboring mutated proteins, exosomes may transport miRNAs and lncRNAs that regulate the expression of crucial signaling molecules, contributing to resistance against targeted therapy. For example, resistance to BRAF inhibitors can be mediated by the content of exosomes from melanoma cells carrying the miR- 222-targeted tumor suppressor gene PTEN [111]. Similarly, exosomes from CMLs bearing BCR-ABL fusion proteins have been shown to induce drug resistance in response to imatinib, a TKI that targets the BCR-ABL fusion protein [112].

### Exosome-mediated immune evasion and resistance to immunotherapy

In this respect, immunotherapy, especially immune checkpoint inhibitors, has shown spectacular success in the treatment of certain cancers, such as melanoma and lung cancer. These therapies work by reactivating the immune system to recognize and attack cancer cells. However, exosomes may contribute to immune evasion and thus to resistance against immunotherapy. Tumor-derived exosomes often carry immune checkpoint molecules such as programmed death-ligand 1 or PD-L1, which interact with the programmed death- 1 receptor on T cells. This interaction dampens T-cell activation, thereby “turning off” the immune response against tumor cells. Cancer cells, via the secretion of exosomes enriched with PD-L1, create an immunosuppressive environment that enables immune escape even in the presence of immune checkpoint inhibitors [113].

This mechanism has been documented in various cancers, including melanoma, lung cancer, and breast cancer, in which high levels of exosomal PD-L1 correlate with poor responses to immunotherapy [114].

Exosomes also carry other immunosuppressive molecules, including TGF-β and IL- 10, which further suppress cytotoxic T-cell and NK cell activities [115]. These exosomes may promote the expansion of Tregs, dampening antitumor immune responses and promoting immune evasion. Clinically, this exosome-mediated immune modulation diminishes responses to immunotherapies, limiting their ability to induce durable responses in cancer patients [116].

The implications of exosome-mediated immune evasion have been extended to biomarker development for predicting the response to immunotherapy. Circulating exosomes carrying PD-L1 or other immunosuppressive molecules could serve as noninvasive biomarkers to predict which patients are less likely to respond to immune checkpoint inhibitors. This would lead to personalized treatment strategies and improve the selection of patients for immunotherapy, with a concomitant potential for better treatment outcomes [117].

### Contribution to tumor heterogeneity and metastasis

Exosome-mediated communication between tumor cells and the tumor microenvironment plays a critical role in driving tumor heterogeneity and metastasis [118]. Bioactive molecules are transferred by tumor-derived exosomes to modulate the behavior of stromal cells, immune cells, and endothelial cells, thus creating an environment that is supportive of the survival and dissemination of tumor cells. Exosomes are implicated in EMT, which is a biological process involving the acquisition of mesenchymal features, such as motility and invasiveness, by epithelial cancer cells. It is closely associated with metastatic potential and resistance to therapy. Cancer cell-derived exosomes from EMT-positive cells contain pro-EMT molecules, including TGF-β, miRNAs, and transcription factors such as Snail and Twist, which induce EMT in recipient cells.

This leads to the selection of a more aggressive and invasive cancer cell population, thereby increasing the possibility of metastasis [119].

Moreover, exosomes facilitate the formation of premetastatic niches by delivering factors that precondition distant organs for the arrival of metastatic cancer cells. These exosomes promote angiogenesis, ECM remodeling, and immune modulation, which together favor the implantation of cancer cells into secondary tumors. Clinically, metastasis mediated by exosomes complicates treatment because metastatic tumors are more refractory to conventional therapies, thus contributing to poor patient outcomes [120]. Exosomes have a crucial role in organ-specific metastasis through the delivery of bioactive molecules that reshape the TME and condition distant organs for cancer cell colonization. Organotropism is mainly determined by molecular cargos such as proteins, lipids, DNA, and non-coding RNAs that regulate metastasis patterns. Exosomal integrins have been identified as metastatic targeting determinants, with lung metastasis associated with integrins α6β4 and α6β1 and αvβ5 integrins targeting liver metastasis [72]. These exosomes trigger pre-metastatic niche formation by fibroblast activation, immune suppression, and angiogenesis that create a permissive metastatic niche. For instance, liver-targeting exosomes interact with Kupffer cells to trigger pro-inflammatory responses while those crossing the blood–brain barrier (BBB) may trigger neuroinflammation to facilitate metastasis to the brain. In addition, exosomes enriched with osteoclast-activating factors facilitate bone metastasis by promoting bone degradation [121]. Since they participate in cancer progression, exosomal markers such as integrins and miRNAs are being explored as predictive biomarkers of metastatic dissemination, and therapeutic strategies against exosome biogenesis, uptake, or molecular content to minimize metastasis risk are being explored [122].

### Potential therapeutic strategies targeting exosomes

Given the involvement of exosomes in therapy resistance, targeting these vesicles indeed offers some promise for overcoming resistance to cancer therapies. Many strategies have targeted the inhibition of biogenesis, secretion by parental cells, and uptake by recipient cells. Optimizing exosome-targeted therapy includes the formulation of innovative strategies that boost their specificity, stability, and therapeutic efficiency. Surface engineering is one of the strategies in which exosome membranes are altered with functional ligands, antibodies, or peptides to improve targeting efficiency. For instance, an anti-CD19-exosome delivery system has been reported to be efficient in targeting central nervous system lymphoma through crossing the blood–brain barrier [123]. Genetic modification is another strategy in which donor cells are transfected with plasmids encoding surface proteins that enhance exosome homing abilities. Such genetically engineered exosomes are effective in delivering targeted siRNA to tumor cells, improving treatment efficacy [124].

Cargo loading is another crucial aspect in the optimization of exosome therapies. Pre-loading strategies, where therapeutic molecules are directly delivered into parent cells before exosome generation, allow for efficient drug packaging. This strategy has been successfully employed in mesenchymal cell-derived exosomes loaded with miR- 29a- 3p for treating renal fibrosis [125]. Post-loading strategies, such as electroporation, sonication, and incubation methods, are employed to load drugs into purified exosomes. For example, sonication has been employed to load chemotherapeutic agents into exosomes for treating glioblastoma, improving drug stability and targeted delivery [126].

For the purpose of enhancing exosome function, surface functionalization techniques take advantage of RGD peptides, antibodies, or folate groups in order to strengthen targeting efficacy. For instance, exosomal miR- 125b- 5p was bioengineered for the purpose of enhancing PD- 1 therapy in colon cancer models, which significantly increased therapeutic responses [127]. Ensuring the purity of exosomes is essential to increase their therapeutic precision. Advanced purification and isolation techniques, including ultracentrifugation, size-exclusion chromatography, and microfluidics, reduce off-target effects and improve the safety of treatment [128]. By an integration of these strategies, researchers are constantly creating exosome-targeted therapies with enhanced precision, stability, and therapeutic efficacy.

## Therapeutic strategies to overcome exosome-mediated resistance

**Fig. 5:**
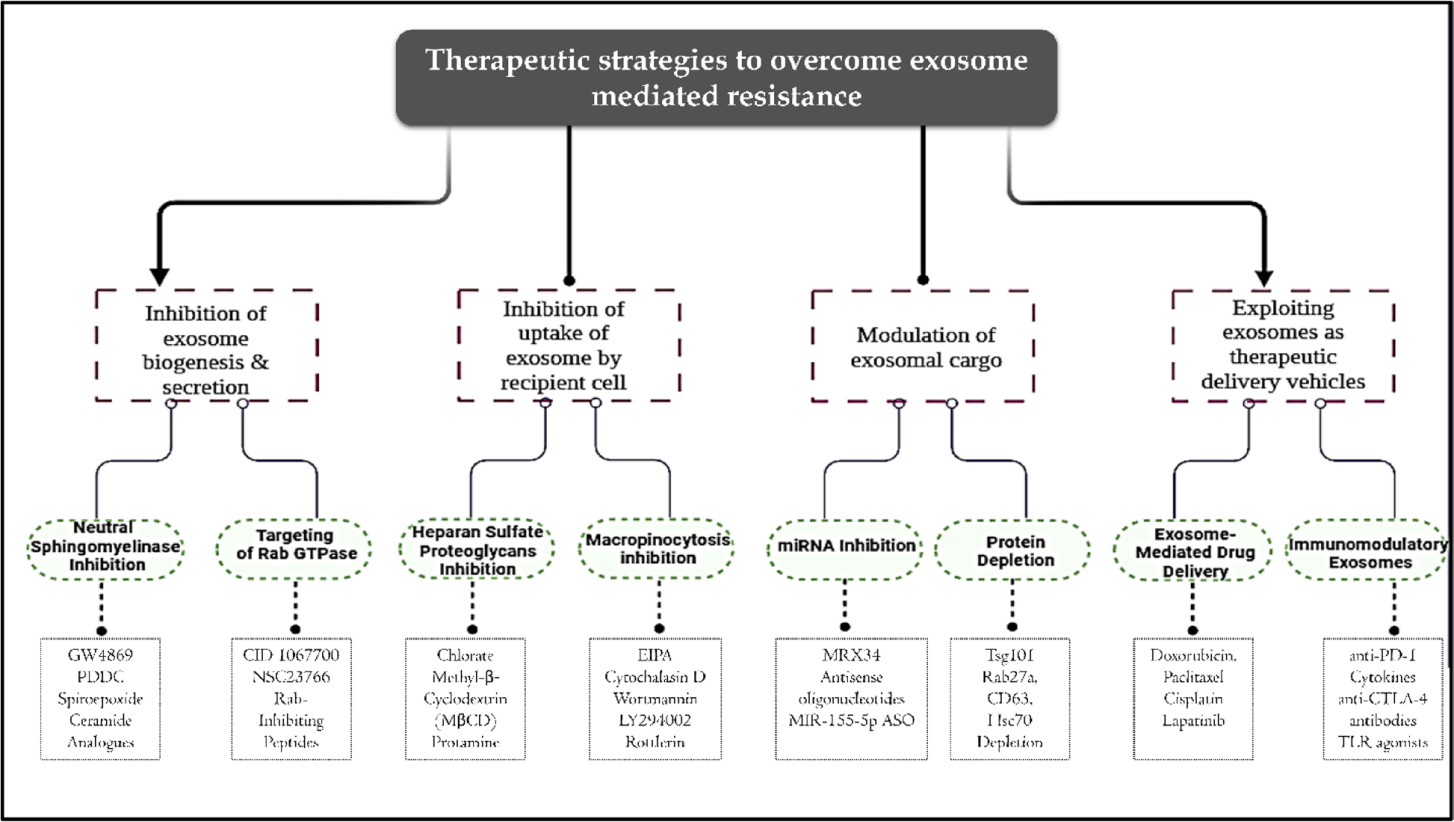
Different strategies to overcome exosome-mediated resistance

Exosome-mediated resistance in cancer has become a formidable challenge in the management of various malignancies. Exosomes are nanosized extracellular vesicles secreted by both tumor and stromal cells and have emerged as important mediators of intercellular communication within the tumor microenvironment [129]. Targeting exosome-mediated resistance has the potential to significantly improve the efficacy of existing therapies, and several novel therapeutic approaches are currently in development. As shown in Fig. 5, several approaches can mitigate exosome-mediated resistance by inhibiting exosome biogenesis, secretion, uptake, and the functional consequences of exosome-mediated communication [130]. Moreover, exosomes are being explored as therapeutic delivery vehicles for anticancer agents, creating a dual-edged approach in the fight against exosome-mediated resistance.

### Inhibition of exosome biogenesis and secretion

Exosome biogenesis is a multistep process initiated by the formation of intraluminal vesicles within multivesicular bodies and culminates in the fusion of MVBs with the plasma membrane, resulting in the release of exosomes into the extracellular space. Targeting the molecular machinery involved in exosome formation and secretion has emerged as a promising strategy to disrupt exosome-mediated resistance [131].

#### Neutral sphingomyelinase inhibition

Neutral sphingomyelinase 2, an enzyme responsible for the hydrolysis of sphingomyelin to ceramide, is considered one of the key regulators of exosome biogenesis. The generation of ceramide is important for inducing the budding of ILVs into MVBs. Inhibition of nSMase2 via the use of certain inhibitors, such as GW4869, has been shown to reduce exosome production in various cancer models [132]. These compounds exert their functions through the inhibition of exosome biogenesis, which, in turn, reduces the intercellular transfer of drug resistance factors such as miRNAs and proteins, thus sensitizing tumor cells to chemotherapy and targeted therapies. This therefore represents a very potent strategy for overcoming resistance in cancers such as breast cancer, where the exosomal transfer of MDR proteins and miRNAs has been implicated in therapeutic failure [133].

#### Targeting of Rab GTPase

Rab GTPases, especially Rab27a and Rab27b, are important regulators of MVB trafficking and exosome secretion [134]. Knockdown or inhibition of Rab GTPases has been shown to impair the release of exosomes, thus limiting the spread of resistance-associated factors within the tumor microenvironment. Small-molecule inhibitors or siRNAs targeting Rab27 have shown efficacy in reducing exosome secretion and sensitizing cancer cells to chemotherapeutic agents in preclinical models [135]. This approach may be particularly effective in cancers, such as pancreatic and ovarian cancer, where exosome-mediated communication represents one of the major drivers of therapy resistance and metastasis.

### Inhibition of the uptake of exosomes by recipient cells

The critical step in exosome-mediated communication involves the uptake of such exosomes by recipient cells. Exosomes may contact target cells through either interactions between ligands and their receptors on the cell membrane or fusion with the plasma membrane and subsequent endocytosis of the exosomes. Tumoral or stromal preclusion of exosomal uptake could impede the transfer of factors associated with resistance and intercept the cross-talk involved in driving resistance to therapy [136].

#### Heparan sulfate proteoglycan inhibition

Heparan sulfate proteoglycan inhibition (HSPGs) on the recipient cell surface are critical for exosome binding and uptake. Inhibition of HSPGs or blockade of their interaction with exosomes decreases exosome uptake. Surfen, an inhibitor of heparan sulfate, was used to block exosome internalization and reduce exosome-mediated transfer of drug resistance in vitro. Analogously, other inhibitors of HSPGs or their receptors could be designed as therapeutic agents that could block the uptake of exosomes and thus potentiate existing cancer therapy [137].

#### Macropinocytosis inhibition

Macropinocytosis, a form of endocytosis, is considered one of the major pathways of exosome uptake in cancer cells. Known inhibitors of macropinocytosis, including amiloride and EIPA (5-(N-ethyl-N-isopropyl)-amiloride), decrease the internalization of exosomes and impair the transfer of resistance factors. Interference with this pathway offers the possibility of blocking exosome-mediated resistance in those tumors where macropinocytosis is a dominant mechanism for exosome uptake [138].

### Modulation of the exosomal cargo

Exosomes carry a wide range of biomolecules, including proteins, miRNAs, and lipids, which contribute to the development of therapy resistance. Modulating the molecular cargo of exosomes to neutralize or block the transfer of resistance-associated factors is an attractive therapeutic strategy.

#### miRNA inhibition

miRNAs, which are carried by exosomes, are implicated at the hub in controlling the expression of genes in recipient cells. Some miRNAs are linked to the pathogenesis of cancer through functions such as facilitating drug resistance and immune evasion and metastasis. In cancer, both miR- 21 and miR- 155 have been detected in many exosomes taken from drug-resistant cancer types, whose action is crucial for regulating cell survival and proliferation pathways [139]. These miRNAs might be targeted by specific antagomirs or antisense oligonucleotides, and inhibitors of miRNAs that block the transfer of resistance signals can restore sensitivity to therapy. Some clinical trials that have used miRNA inhibitors such as anti-miR- 21 are currently in progress in various cancers, hence providing a promising avenue for escape from exosome-mediated resistance [140].

#### Protein depletion

Resistance-conferring exosomal proteins can either be drug efflux pumps, such as P-glycoprotein, or oncogenic receptors, such as HER2, thereby providing a way of conferring resistance both by facilitating the efflux of drugs and/or inducing prosurvival signals in recipient cells. Approaches that deplete or neutralize exosomal proteins may be instrumental in reversing resistance mechanisms [141]. For instance, therapeutic blockade of exosomal HER2 or P-glycoprotein using specific monoclonal antibodies should, in theory, result in a reduced magnitude of resistance to both targeted therapies and chemotherapy [142].

### Exploiting exosomes as therapeutic delivery vehicles

While exosomes contribute to therapy resistance, they display unique properties that make them ideal candidates for drug delivery. Owing to their natural ability to carry bioactive molecules, along with their excellent biocompatibility, exosomes are attractive vehicles for the direct delivery of various therapeutic agents, including small molecules, nucleic acids, and proteins, to tumor cells.

#### Exosome-mediated drug delivery

Engineered exosomes can be loaded with either chemotherapeutic drugs or siRNAs targeting key resistance pathways. These engineered vesicles utilize tumor-targeting ligands on the surface to selectively deliver their cargo into cancer cells, thereby reducing off-target effects and increasing drug efficacy [143]. For example, exosomes loaded with paclitaxel or doxorubicin improved drug delivery and tumor suppression in preclinical models of breast and lung cancer. Similarly, exosomes carrying siRNAs that target oncogenes such as KRAS or EGFR hold promise for overcoming resistance to targeted therapy [144].

#### Immunomodulatory exosomes

Exosomes can also be designed to carry immune-activating molecules, including tumor-associated antigens or immune checkpoint inhibitors, to augment antitumor immune responses. A typical example of this includes exosomes that are loaded with inhibitors against programmed death-ligand 1 or cytotoxic T lymphocyte-associated antigen 4, which increase the efficiency of immunotherapy in resistant cancers. In addition, exosomes derived from TAA-expressing dendritic cells induce potent T-cell responses and represent a new approach in cancer immunotherapy [145].

### Combined therapies targeting exosomes and conventional treatments

Exosome-mediated resistance remains complex; hence, combination therapies targeting exosome pathways, in addition to conventional treatments, are promising options. Table 1 provide the summary of mechanism and applications of the inhibitors used to target exosome biogenesis, secretion, and uptake. Combining exosome inhibitors with chemotherapy or targeted therapy may increase drug sensitivity by decreasing the ability of tumor cells to develop resistance. In contrast, combining exosome-targeting strategies with immunotherapy may enhance immune responses through the blockade of exosome-mediated immune evasion [146].

**Table 1:** Summary of the mechanisms and applications of the inhibitors used to target exosome biogenesis, secretion, and uptake

Category	Inhibitor	Mechanism	Applications	References
**Inhibitors of exosome biogenesis**	**GW4869**	Inhibits neutral sphingomyelinase (nSMase), blocking ceramide biosynthesis crucial for exosome formation	Reduces exosome release in cancer and neurodegenerative disease studies	[147]
	**Manumycin A**	Inhibits farnesyltransferase, interfering with RAS signaling involved in exosome biogenesis	Effective in reducing tumor-derived exosome production	[148]
	**Y27632**	Targets Rho-associated protein kinase (ROCK), affecting cytoskeletal dynamics essential for exosome formation	Studied in cancer and fibrosis models	[149]
**Inhibitors of exosome secretion**	**Calpeptin**	Inhibits calpain, a protease involved in vesicle trafficking and cytoskeletal rearrangement	Investigated in cancer models to prevent tumor progression	[150]
	**Bisindolylmaleimide I (BIM I)**	Inhibits protein kinase C (PKC), reducing vesicle release from cells	Used in preclinical studies to suppress exosome secretion in inflammatory conditions	[151]
	**Cannabidiol and SMR peptides**	It has been found to affect mitochondrial functions by reducing the expression of STAT3 and prohibitin, both of which positively regulate cell proliferation, and hence is a possible agent to sensitize chemoresistant cells to chemotherapy drugs	Primarily used in experimental setups to block general vesicle secretion. SMR peptides blocked EV release in the cell lines MCF- 7 and MDA-MB- 231	[152, 153]
**Inhibitors of exosome uptake**	**Dynasore**	Inhibits dynamin, a GTPase involved in endocytosis and membrane trafficking	Reduces the uptake of tumor-derived exosomes in cancer studies	[154]
	**Chlorpromazine**	Inhibits clathrin-mediated endocytosis by blocking clathrin-coated pit formation	Used to block exosome uptake in various cellular studies	[155]
	**Amiloride**	Inhibits micropinocytosis by targeting Na +/H + exchangers	Reduces exosome uptake in cancer and viral infection models	[156]
**Emerging exosome inhibitors**	**Tipifarnib**	Farnesyltransferase inhibitor targeting exosome production in RAS-driven cancers	Investigated for therapeutic potential in cancer	[157]
	**Neticonazole**	Reduces ceramide synthesis, thereby inhibiting exosome formation	Explored in cancer and other diseases	[158]
	**Brefeldin A**	Disrupts the Golgi apparatus, impeding vesicular trafficking	Investigated for inhibiting general vesicle-mediated processes	[159]

## Comparison of exosome-based drug delivery vs. nanoparticle-based delivery systems

Exosome-based drug delivery systems are gaining great attention due to their natural origin, biocompatibility, and targeting capability. However, traditional nanoparticle-based systems like liposomes, polymeric nanoparticles, and metallic nanoparticles have long been established in drug delivery. Table 2 provides comprehensive comparison of exosome based and nanoparticle-based drug delivery systems, focusing on their respective efficacy and safety profiles.

**Table 2:** Exosome-based and nanoparticle-based drug delivery systems: Safety and efficacy perspective

	**Exosome-based delivery**	**Nanoparticle-based delivery**
**Aspect related to efficacy**		
**Drug loading efficiency**	Limited intrinsic loading capacity; requires engineering	High loading efficiency with tunable designs
**Targeting ability**	Superior targeting via natural homing properties	Enhanced targeting through ligand modifications
**Therapeutic potential**	Emerging applications in regenerative medicine and immunotherapy	Proven efficacy in cancer, infectious diseases, and vaccines
**Delivery efficiency**	Efficient delivery in biological environments	Requires advanced coating strategies for sustained release
**Aspect related to safety**		
**Biocompatibility**	High biocompatibility due to natural origin	Requires surface modification for improved biocompatibility
**Immune response**	Low immunogenicity; mimics natural cell processes	Potential immune activation and inflammation risks
**Stability**	Moderate stability; prone to degradation under stress	Highly stable with engineered coatings and polymer designs
**Toxicity risk**	Minimal toxicity; derived from endogenous cellular material	Potential toxicity depending on material composition

## Future directions

Exosome research is confronted with some key challenges like heterogeneity, large-scale production issues, and the demand for accurate profiling techniques. The heterogeneity of exosomes due to variations in cell origin, molecular content, and size makes their characterization and their use in clinics more complicated. New methods like single-exosome profiling using nanoflow cytometry and nanoimaging technologies are increasing accuracy. Large-scale production is also challenging as conventional isolation methods yield low reproducibility and low purity. New options like 3D cell culture systems, bioreactors, and engineered exosome production are being explored to boost scalability and reproducibility. Single-exosome profiling techniques like droplet microfluidics and surface-enhanced Raman scattering (SERS) are enabling detailed molecular information at a single exosome level. Exosome barcoding techniques like ExoID and nanoparticle barcoding offer high-throughput analysis through exosome tagging to permit multiplex detection to increase diagnosis accuracy and biomarker discovery. All such advancements play a key role in overcoming current hurdles and enabling exosome-based therapeutics and diagnostics.

## Conclusion

Exosomes have emerged as regulators of cellular conversion and play critical roles in disease development and chemotherapy resistance in many malignancies, including cervical carcinoma. Biogenesis modalities for generating them include sophisticated pathways for packaging and delivery of a range of molecular contents, such as proteins, RNAs, and lipids. These vesicles can not only mold tumor behavior but also, through the efflux of drugs, modulate apoptosis, EMT, and the suppression of immune function, can mold a changing and dynamic microenvironment in an emerging tumor. The multifaceted role of exosomes in therapy resistance involves the export of drugs out of cells and the regulation of survival signals, which interferes with the efficacy of drugs. In addition, EMT can be triggered through exosomal contents, and metastasis and heightened tolerance toward traditional therapies can be stimulated through these processes. In addition, through immune manipulation, evasion of immune surveillance in cancer cells is facilitated through exosomes, increasing the effectiveness of therapy.

Clinically, exosomes have dual potential both for use as markers for the prognosis and diagnosis of cancer and for overcoming obstacles in therapy. Their predictive role in therapeutic efficacy is a strong marker for their use in diagnostics, but converting such a potential into effective therapeutic interventions is a challenge yet to be overcome. Existing studies target the development of exosome inhibitors, engineered exosomal therapies, and combination therapies to overcome exosome-related resistance. Exosome engineering and future technology have therapeutic potential for personalized therapy, with therapies having the ability to target specific individual patient profiles of exosomes and, in a broader picture, for enhanced therapeutic efficacy and therapeutic success in terms of patient outcomes.

## Data Availability

No datasets were generated or analysed during the current study.
